# Prevalence of germline TP53 variants among early-onset breast cancer patients from Polish population

**DOI:** 10.1007/s12282-020-01151-7

**Published:** 2020-09-04

**Authors:** Emilia Rogoża-Janiszewska, Karolina Malińska, Bohdan Górski, Rodney J. Scott, Cezary Cybulski, Wojciech Kluźniak, Marcin Lener, Anna Jakubowska, Jacek Gronwald, Tomasz Huzarski, Jan Lubiński, Tadeusz Dębniak

**Affiliations:** 1 Department of Genetics and Pathology, International Hereditary Cancer Center, Pomeranian Medical University, Szczecin, Poland; 2 School of Biomedical Sciences and Pharmacy, University of Newcastle, Newcastle, NSW Australia; 3 Department of Molecular Medicine, NSW Health Pathology-Hunter, Newcastle, NSW Australia

**Keywords:** TP53, Early-onset breast, Cancer, Li-fraumeni, Mutation

## Abstract

**Background:**

The objective of this study was to determine spectrum and prevalence of germline mutations in *TP53* gene among Polish women with early-onset breast cancer (BC), which has not been determined until now.

**Methods:**

A cohort of 100 females with BC diagnosed ≤ 30 years of age and with a positive family history of cancer was used as a discovery cohort. 1880 women with BC ≤ 45 years old and a control group of 2000 healthy women were genotyped as a replication phase of this study.

**Results:**

Four heterozygous pathogenic missense mutations were detected in a group of 100 patients with early-onset breast cancer. On the basis of software prediction and available literature data, all these variants were defined as pathogenic. None of these *TP53* variants were detected among 1880 breast cancer patients and 2000 healthy controls. No large mutations were found among early-onset cases using MLPA reaction.

**Conclusion:**

Germline pathogenic *TP53* variants were found in 4% early-onset Polish BC patients. No founder mutations were identified in Polish population. To improve the treatment and surveillance screening, the search for germline TP53 pathogenic variants is recommended for all female BC cases diagnosed ≤ 30 years old.

**Electronic supplementary material:**

The online version of this article (10.1007/s12282-020-01151-7) contains supplementary material, which is available to authorized users.

## Introduction

Breast cancer is the most frequent cancer, affecting 2.1 million women annually, and causes the greatest number of cancer-related deaths among women. In 2018, it was estimated that 627,000 women died from breast cancer—which equates to approximately 15% of all cancer deaths among women. Breast cancer incidence is higher among women in more developed regions, and increasing in nearly every region globally [1].

In Poland, breast cancer accounts for about 23% (18,615 cases) of 81,620 cases of malignant neoplasms in women annually. The incidence among premenopausal women (20–49 years) has increased by approximately 1.7 times over the past three decades. In 2016, 103 cases of breast cancer were reported in women who are 29 years old or younger, and 401 cases diagnosed in women who are 34 years of age or younger [2].

The molecular basis of the vast majority of early-onset BC cases remains unknown. *BRCA *mutations accounts for 10–20% of women with early-onset breast cancer (defined as breast cancer diagnosed under age 40 years) [3]. Mutations in other breast cancer susceptibility genes such as *CDH1, PTEN, STK11, CHEK2, PALB2* and others, may explain up to 4% of early-onset cases [4].

*TP53* pathogenic germline variants are observed in a high percentage of cancers diagnosed at a young age and correlate with an unfavorable prognosis. BC associated with pathogenic germline *TP53* is the most frequent neoplasm among female *TP53* mutation carriers (25.5% of all tumors and 80% of all cancers in the 16–45 year-age women group [5, 6]. *TP53* gene mutations are present in up to 5–7% of all early-onset BC cases diagnosed under 30 years and in 2–6% of BC patients younger than 35 [7, 8]. The incidence of de novo mutations in *TP53* is somewhere between 7 and 20% [9]. Inherited *TP53* gene mutations increase the risk of numerous cancer types, including breast cancer, leukemia, sarcomas, central nervous system (CNS) tumors, and adrenal cortical cancer [as part of Li-Fraumeni syndrome (LFS)] [10]. The most frequent cancer types observed in carriers of *TP53* pathogenic variants were breast cancer (24–31.2%), soft tissue and bone sarcoma (11.6–17.8%), followed by adrenocortical carcinoma (6.5–9.9%), and brain tumors ( 3.5–14%) [7, 10–13]. *TP53* (tumor protein 53 gene) is a tumor suppressor, which controls growth and cell division. It protects cells against genome changes resulting from DNA damage by suppressing proliferation or activating apoptosis [14]. The most common mutations in the total spectrum of changes in the *TP53* gene are point missense mutations (75%), that involve the DNA segment encompassing exons 5–8 [15, 16]. This segment determines the biological activity of the protein. Other germline variants observed in *TP53* are frameshift insertions and deletions (9%), nonsense (7%) and silent mutations (5%) [17]. However, internationally, the incidence of large mutations in *TP53* is estimated to be around 10% [17–19]. People with a heterozygous germline variant in *TP53* have a 50% risk of developing cancer under 31 years of age and almost 100% risk of developing a malignancy before 70 years of age [20].

The objective of this study was to determine the spectrum and prevalence of germline pathogenic variants in *TP53* among Polish women diagnosed with early-onset BC, which has not previously been assessed in the Polish population.

## Patients and methods

### Study groups

Two non-overlapping groups of patients with breast cancer were examined for this study:

- A cohort of 100 unrelated patients diagnosed with early-onset breast cancer ≤ 30 years of age (range of 16–30 years; mean 26 years) was used as a discovery cohort to detect germline *TP53* variants. All patients had a positive family history of cancer: 72 of these 100 families fulfilled the LFS Chompret criteria, with the occurrence of breast cancer among first or second-degree relatives of a proband affected with early-onset BC. The remaining 28 of these 100 families were characterized by the occurrence of another tumors (colorectal, gastric, prostate, ovarian, pancreas, melanoma), which are not found in the spectrum of cancer characteristic of classical LFS among first or second-degree relatives of the early-onset patients.
  The patients were selected from a registry of 25,000 breast cancer cases housed at the Hereditary Cancer Center in Szczecin: out of 448 based on the age of onset of breast cancer (≤ 30 years), out of 388 early-onset cases tested negative for a pathogenic variants in *BRCA1* or *BRCA2*.
- A cohort of 1880 patients with breast cancer diagnosed ≤ 45 years of age (range 19–45 years; mean 39 years), who were diagnosed between 2008 and 2013 at 18 different hospitals in Poland. All patients diagnosed with invasive breast cancer at participating centers were eligible to participate and were unselected for family history. The patient participation rate was 76.1%. Patients from this group were also selected with respect to a negative result for screening tests for gene predisposing to a high risk of developing breast cancer—*BRCA1/2*(-). These patients were used as a replication cohort that allowed the estimation of the frequency of pathogenic variants detected at an earlier stage using TaqMan assays.

Clinicopathological features of the cases are presented in Table 1.

**Table 1:** Clinicopathological features of BC patients

Characteristics	Discovery cohort (*n* = 100)	Validation cohort (*n* = 1880)
Age at diagnosis		
− 20	5 (5%)	1 (0.05%)
21–30	95 (95%)	111 (5.9%)
31–40		804 (42.7%)
41–50		964 (51.3%)
Sex		
Female	100 (100%)	1880 (100%)
Male	0	0
Race		
White (Slavic) population)	100 (100%)	1880 (100%)
Hormone receptor status		
ER	33 (33%)	695 (37%)
PgR	28 (28%)	752 (40%)
HER2	21 (21%)	211 (11.2%)
Triple negative	9 (9%)	162 (8.6%)
Unknown	9 (9%)	60 (3.2%)
Status		
Alive	88 (88%)	1710 (91%)
Dead	12 (12%)	170 (9%)
Histopathological type		
Ductal carcinoma	54 (54%)	950 (50.5%)
Lobular carcinoma	4 (4%)	105 (5.6%)
Medullary carcinoma	3 (3%)	77 (4.1%)
DCIS	2 (2%)	55(2.9%)
Others	10 (10%)	130 (6.9%)
Unknown	27 (27%)	563 (29.9%)

### Controls

The control group consisted of 2000 healthy women (age range of 20–100; mean age of 53).

The healthy adults were deemed as having a negative cancer family history (first- and second-degree relatives included). These women were diagnosed in Szczecin between 1996 and 2004 and were part of a population-based study of the 1.5 million residents of West Pomerania (northwest Poland) to identify familial aggregations of malignancies among first and second-degree relatives from 1.258 million residents (87%) who were registered with the West Pomeranian Regional Health Foundation. The patient participation rates exceeded 75%.

During the interview, the goals of the study were explained, informed consent was obtained, genetic counseling was given and a blood sample taken for DNA analysis. All patients and control subjects are of European ancestry and ethnic Poles.

## Methods

### Discovery phase

DNA was isolated from blood taken from the 100 participants ≤ 30 years of age using standard methods. Peripheral blood leukocytes were isolated and subsequent DNA extraction was undertaken in the Department of Genetics and Pathology in Szczecin. To identify germline mutations in the *TP53*, Sanger sequencing was performed and MLPA was used to screen for larger insertions or deletions.

#### Sanger sequencing

All protein coding fragments of *TP53* (exons 2–11) were examined by the Sanger sequencing. The sequences of the primers are presented in electronic supplementary material (Supplementary Table 1). The sequencing reaction was performed using a BigDye Terminator v3.1 Cycle Sequencing Kit (Life Technologies). Sequencing products were analysed using a genetic analyzer ABI Prism 3500XL (Life Technologies). All *TP53* sequences were compared to the NCBI reference sequence (RefSeq) reported in GenBank: NM_000546.5. The complete region of *TP53* gene was amplified and sequenced in both forward and reverse directions. If a pathogenic mutation was detected, the DNA sample from a second patient was resequenced for confirmation.

#### Multiplex ligation-dependent probe amplification (MLPA)

MLPA reaction was undertaken using the SALSA MLPA kit P056 (MRC Holland, Amsterdam, The Netherlands). MLPA is used for study on both hereditary disorders and tumours.

#### In silico prediction models

All detected variants were checked in appropriate databases and algorithms to assess their potential pathogenicity. Gene variants were submitted to the following in silico prediction models:

- Mutation taster—that includes all publicly available single-nucleotide polymorphisms (SNPs) and indels from the 1000 Genomes Project2 as well as known disease variants from ClinVar3 and HGMD Public4. Test results are the evaluated by a naive Bayes classifier2, which predicts the disease potential [21].
- ClinVar—ClinVar processes submissions reporting variants found in patient samples, assertions made regarding their clinical significance, information about the submitter, and other supporting data. The alleles described in submissions are mapped to reference sequences, and reported according to the HGVS standard [22].
- PROVEAN (protein variation effect analyzer)—predicts whether an amino acid substitution or indel has an impact on the biological function of a protein [23].
- PolyPhen-2(polymorphism phenotyping v2)—automatic tool for prediction of possible impact of an amino acid substitution on the structure and function of a human protein [24].
- Align-GVGD—The program allows you to analyze your own protein sequence (in FASTA format), or choose from our small but growing library of matches [25].
- IARC TP53 Database—compiles various types of data and information on human *TP53* gene variations related to cancer. Data are compiled from the peer-reviewed literature and from generalist databases [17].

### Association phase

Changes that showed the strongest functional relationship with tumor pathogenesis (analysis used algorithms and databases) were selected for verification in the second stage of the project—association studies. These studies were carried out using real-time PCR.

### Real-time PCR

The *TP53* variants detected during discovery phase were genotyped using a TaqMan assay (Applied Biosystems/Life Technologies) and the LightCycler Real-Time PCR 480 system (Roche Life Science). The primer and probe sequences were avaiable upon request. Laboratory technicians were blinded to case–control status. The overall genotyping call rate was 99.3%.

### Statistical analysis

All genotype comparisons between cases and controls were performed using Chi-square test. OR value was calculated for each comparison together with their 95% CI.

## Results

Four heterozygous pathogenic germline mutations were detected in a group of 100 patients with early-onset breast cancer: (1) c.844C > T (p.Arg282Trp, rs28934574), (2) c.818G > A (p.Arg273His, rs28934576), (3) c.733G > A (p.Gly245Ser, rs28934575), (4) c.659A > G (p.Tyr220Cys, rs121912666). On the basis of available literature data, informations in databases and in silico analysis revealed these variants to be “missense” variants defined as pathogenic/likely pathogenic. One recurrent common missense variant- c.215C > G (p.Pro72Arg, rs1042522) was also identified (Table 2). Additionally, four synonymous, three 5′UTR and twelve intronic alterations were found (Table 3).

**Table 2:** Missense TP53 mutations detected in Polish early-onset BC patients

Location	SNP	Cons e que nce	IARC TP53 Databas e (TA Clas s)	Mutation Tas te r	ClinVar	PROVEAN	PolyPhe n-2	AGVGD
c.844C > T	rs28934574	p.Arg282Trp	Non-functional	Prediction disease causing	Pathogenic	− 7.65 Deleterious	1.0 Probably damaging	Class C65
c.818G > A	rs28934576	p.Arg273His	Non-functional	Prediction disease causing	Pathogenic/likely pathogenic	− 4.775 Deleterious	0.999 Probably damaging	Class C25
c.733G > A	rs28934575	p.Gly245Ser	Non-functional	Prediction disease causing	Pathogenic	− 5.877 Deleterious	0.998 Probably damaging	Class C55
c.659A > G	rs121912666	p.Tyr220Cys	Non-functional	Prediction disease causing	Pathogenic	− 8.592 Deleterious	0.949 Probably damaging	Class C65
c.215C > G	rs1042522	p.Pro72Arg	Not available	Prediction polymorphism	Benign	− 0.186 Neutral	0.217 Benign	Class C0

**Table 3:** TP53 non-pathogenic variations identified in Polish early-onset breast cancers

**Variant**	**Exon/Intron**	**Amino acid**	**rs number**	**Effect**	**Number of cases**
c.74 + 38C > G	Intron 2	–	rs1642785	5′UTR variant	18
c.75-40G > A	Intron 2	–	rs372756865	5′UTR variant	1
c.75-48 T > C	Intron 2	–	rs1295353442	5′UTR variant	5
c.97-29C > A	Intron 3	–	rs17883323	Intronic	2
c.96 + 25ACCTGGAGGGCTGGGG	Intron 3	–	rs59758982	Intronic	26
c.97-29C > A	Intron 3	–	rs17883323	Intronic	2
c.560-40A > G	Intron 5	–	rs377059569	Intronic	1
c.618G > A	Exon 6	p.Leu206 =	rs142813240	Synonymous	3
c.639A > G	Exon 6	p.Arg213 =	rs 1,800,372	Synonymous	1
c.672 + 62A > G	Intron 6	–	rs1625895	Intronic	7
c.782 + 72C > T	Intron 7	–	rs12947788	Intronic	8
c.782 + 92 T > G	Intron 7	–	rs 12,951,053	Intronic	8
TP53_g.14032G > A	Exon 9	p. Lys320 =	–	Synonymous	2
c.993 + 12 T > C	Intron 9	–	rs1800899	Intronic	1
17:7673445G > T	Intron 9	–	–	Intronic	1
c.1077A > G	Exon 10	p. Pro359 =	rs539224556	Synonymous	1
c.1100 + 44C > T	Intron 10	–	rs905346274	Intronic	1
c.1100 + 30A > T	Intron 10	–	rs17880847	Intronic	3
c.1101-49C > T	Intron 10	–	rs17881850	Intronic	3

**Fig. 1:**
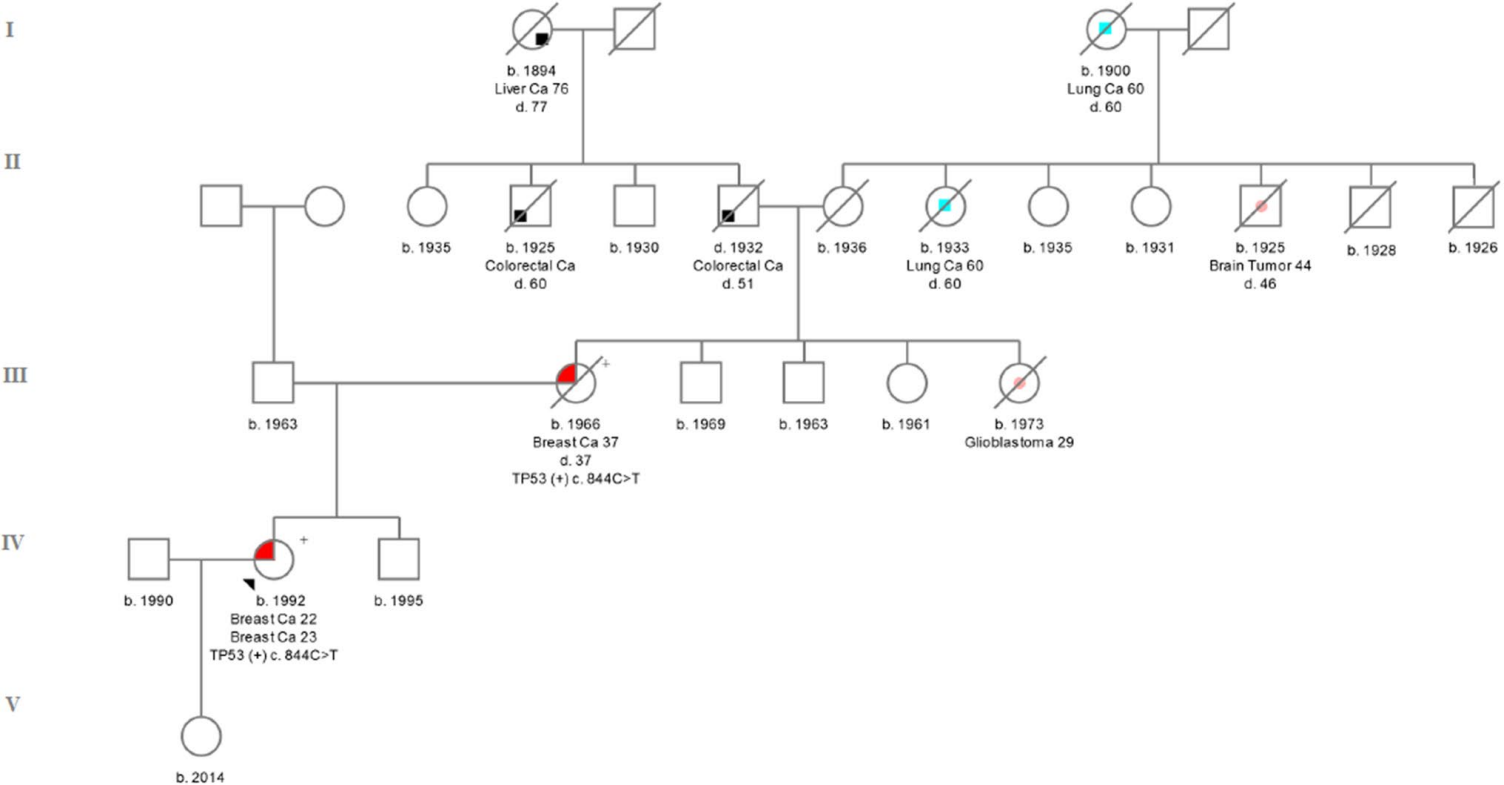
Pedigree of the family with c.844C > T detected variant. ( +) mutation positive, (−) mutation negative

*TP53 c.844C > T (rs28934574)* was localized in exon 8. This variant converts the amino acid arginine (CGG) to tryptophan (TGG) in codon 282-**p. Arg282Trp**. 844C > T, was initially reported as a founder mutation in the French-Canadian population [26]. The IARC database describes this germline mutation in 65 cases [17]. According to the databases, the incidence of this variant among the European population is 1/135 368 Europeans (0.00001) [27]. It was found that the change in R282W is associated with a much earlier age of onset of the first tumor [28]. The patient in whom it was detected developed breast cancer [ER( +), PR(−), HER2 (3 +)] at the age of 22 during pregnancy was diagnosed. The proband’s mother had BC at the age of 37 [ER(−), PR(−), HER2(3 +)]. Family history of the proband was presented on Fig. 1.

**Fig. 2:**
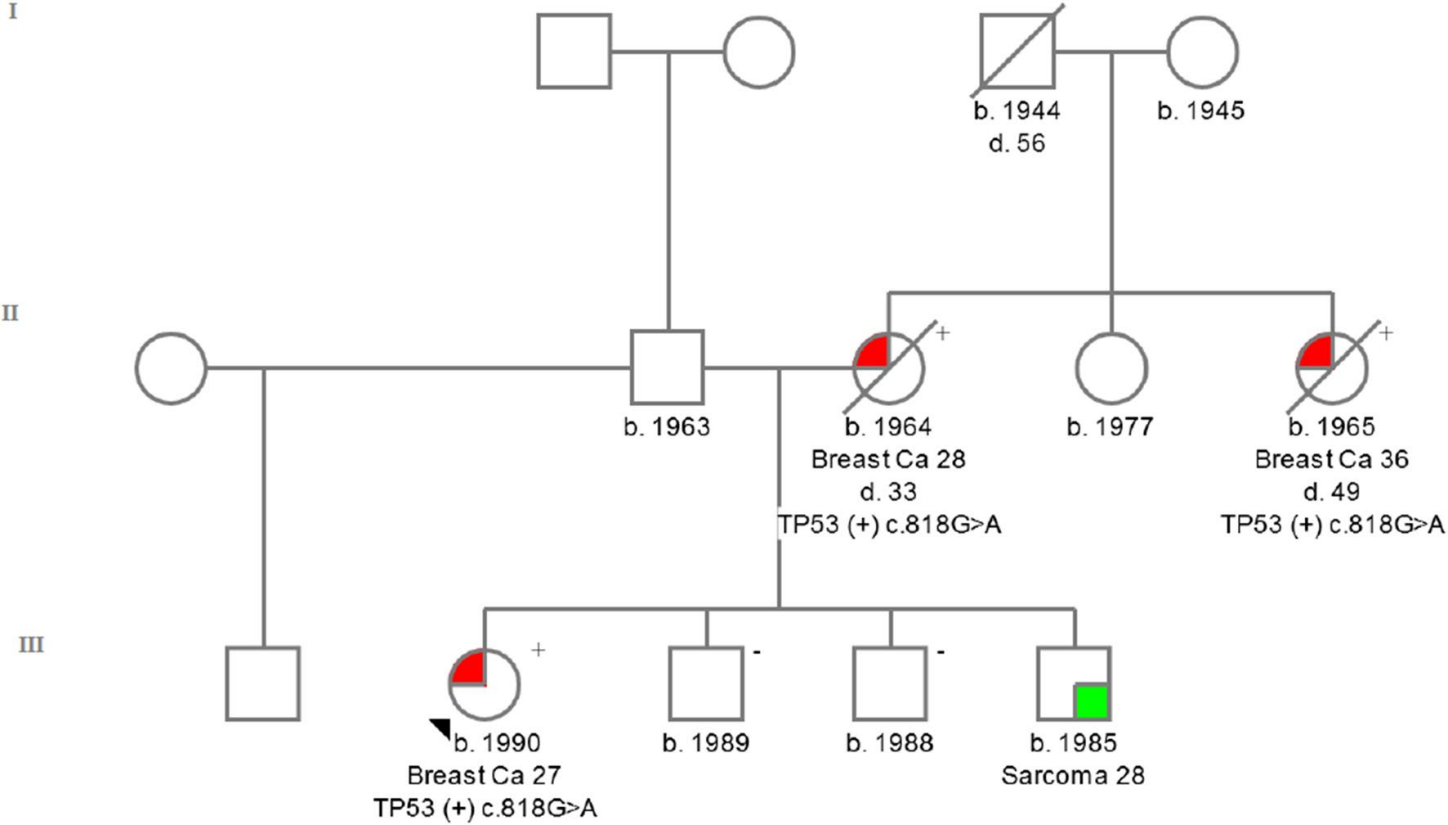
Pedigree of the family with c.818G > A detected variant. ( +) mutation positive, (−) mutation negative

*TP53 c.818G > A (rs28934576)* was detected in exon 8. This change causes the conversion of the amino acid arginine (CGT) to histidine (CAT) in codon 273-**p. Arg273His**. The patient in whom this change was identified developed breast cancer at the age of 27 years [ER(−), PR(−), HER2(3 +)]. The c.818G > A variant is the second most commonly reported mutation in the *TP53* gene in the COSMIC database [29]. The incidence of this variant among the European population is 1/1006 (0.001) Europeans [27]. The IARC database describes this germline mutation in 97 carriers [23]. The family history of the proband was presented on Fig. 2.

*TP53 c.733G > A (rs28934575)* identified in exon 7, converts the amino acid glycine (GGC) to serine (AGC) in codon 245—**p. Gly245Ser**. The patient with *TP53* c.733G > A change was diagnosed at the age of 26 years with ductal breast cancer [ER(−), PR(−), HER2(3 +)].

**Fig. 3:**
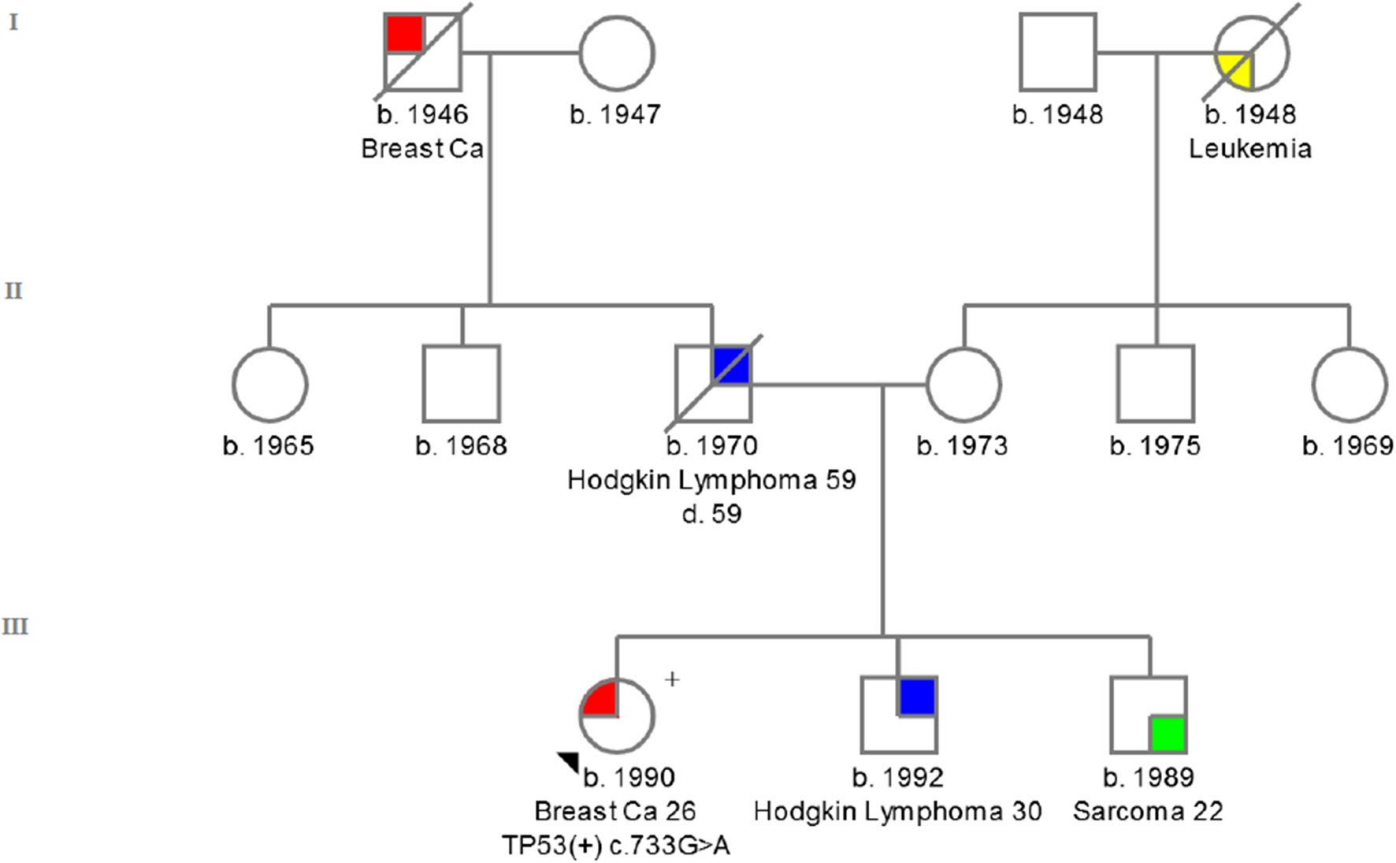
Pedigree of the family with c.733G > A detected variant. ( +) mutation positive, (−) mutation negative

The incidence of this variant in the general population is 1/125 568 (0.00001) [27]. The IARC database describes this germline variant in 77 cases [17]. The family history of the proband was presented in Fig. 3.

**Fig. 4:**
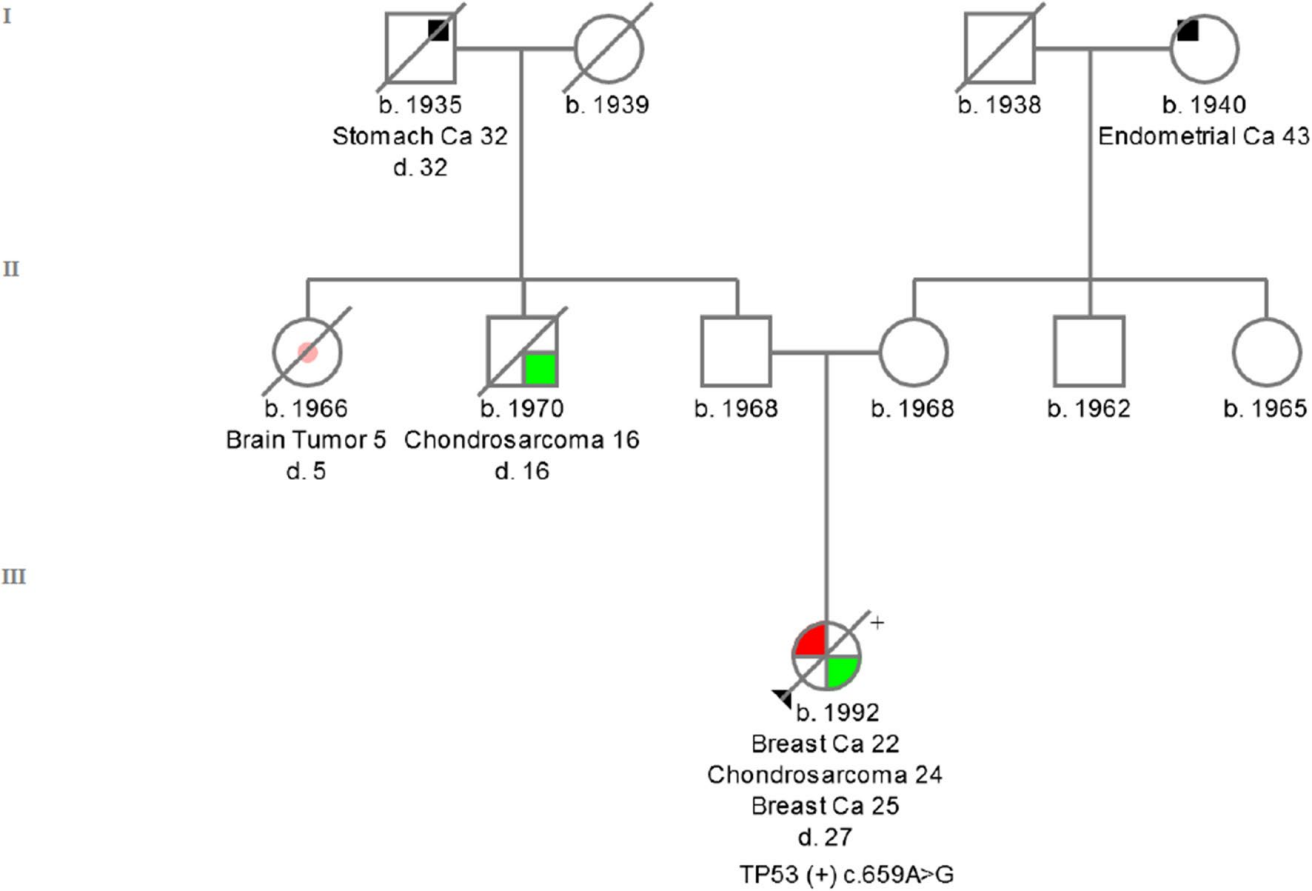
Pedigree of the family with c.659A > G detected variant. ( +) mutation positive, (−) mutation negative

*TP53 c. 659A > G (rs121912666)* is located in exon 6**.** This missense mutation converts the amino acid tyrosine (TAT) to cysteine (TCT) in codon 220 of the *TP53* protein—**p.Tyr220Cys**. TP53 c. 659A > G occurs in families with the LFS phenotype. At the age of 22, the carrier was diagnosed with ductal BC; 2 years later, she developed chondrosarcoma on the right scapula, histological grade G2, and at the age of 26, a ductal BC [ER( +), PR(−), HER2 (1 +)]. The patient died at the age of 27. The incidence of this variant among the European population is 1/135 358 (0.00001) Europeans [27]. The IARC database describes this variant among 50 individual carriers [17]. The family history of the proband was presented on Fig. 4.

### Large mutations

No DNA alterations were detected among the 100 early-onset BC patients using MLPA reaction.

### TaqMan analysis

None of *TP53* variants (c.818G > A, c.733G > A, c.659A > G, c.844C > T) were detected among 1880 breast cancer patients or the 2000 healthy controls. The *TP53* c.844C > T variant was detected in 1 out of 1880 BC cases. However, after genetic counselling, it turned out that this patient is a first degree relative of a proband (Fig. 1).

A non-pathogenic missense variant c.215C > G was detected in 747 out of 1880 BC cases (39.7%) and 797 out of 2000 controls (39.8%). The difference in allele frequency was not statistically significant (OR − 0.995; *p*-value − 0.967).

## Discussion

The aim of this study was to determine the spectrum and prevalence of germline pathogenic variants in *TP53* among early-onset breast cancer patients from the Polish population. We found that germline *TP53* pathogenic variants can be found in 4% of Polish early-onset BC patients with a positive cancer family history. None of these four variants appeared to be recurrent. No large insertions or deletions were found and thus are extremely rare. Only one of the four families meets classical LFS criteria. Our research confirmed the frequency of pathogenic variants in *TP53* as determined by others. All detected variants were missense mutations and have previously been described in other reports [30–32].

In The Netherlands, Bakhuizen et al. [33] revealed in 370 women diagnosed with BC < 30 years of age not carrying any deleterious variants in *BRCA1/2* eight (2.2%) *TP53* germline pathogenic variants. In women without a family history suggestive of LFS or a personal history of multiple LFS-related tumors, the *TP53* pathogenic variant frequency was < 1%. In the Mexican population, five pathogenic variants (9.4%) were found in women with invasive ductal and lobular carcinomas, younger than 36 years of age and *BRCA1/2* negative. All patients had a familial history suggestive of LFS. One of the variants (c.844C > T) was initially reported as a founder mutation in the French-Canadian population [34] and was detected in our study. Among 2 out of 133 Taiwanese patients (median age—44) were found to carry the germline pathogenic variant p.G245S and p.R248Q in *TP53*, which results in the defective function of *TP53*. 94 out of 133 patients had positive family history of cancer. The mutation p.G245S was also detected in our study. Both patients with a TP53 mutation were diagnosed as Li-Fraumeni syndrome [30].

Rashid M.U. et al. assessed the prevalence of *TP53* variants in 105 early-onset BC patients from Pakistan (negative for *BRCA1/2* pathogenic variants). The study group included 67 women diagnosed with BC ≤ 30 years of age with no family history of breast or ovarian cancer and 38 women diagnosed with BC ≤ 40 years with one or more first- or second-degree relatives with breast or ovarian cancer. One rare deleterious variant (c.499-500delCA) in *TP53* was identified in a 28-year-old BC patient with a negative family history [35].

In Canada, 6 (21.4%) out of 28 women with early-onset BC (diagnosed < 30 years) and a positive family history of cancer were found to carry pathogenic *TP53* variants—c.708C > A, IVS03-11C > G, c.524G > A, c.818G > A, c.743G > A). Five from the six mutation-positive patients (83%) met Chompret criteria, and one (7.7%) did not meet any current criteria for LFS. They have previously tested negative for mutations in *BRCA1/2* genes [36].

In the United States, three variants (p.G245S, p.C277stop, p.R175H) in *TP53* were detected among 3/300 (1%) breast cancer patients (regardless of age) with positive family history. They came from any region in the United States and self-defined ancestry. All detected pathogenic variants were associated with LFS/LFL syndrome. One of the variants in this study was identified in the current study (p.G245S) [7].

From an Australian population-based series of invasive BC, that included, (a) very early-onset BC cases: 52 women diagnosed under the age of 30 years, unselected for family history and; (b) early-onset disease, 42 women diagnosed under the age of 39 years with positive family history breast or ovarian cancer. In the first subgroup, 2 pathogenic *TP53* variants were identified (4%)—G13203A, 7568-12905del. Neither had a family cancer history that met the criteria for Li-Fraumeni syndrome. In the second subgroup of 42 patients, 3 (7%) carried a pathogenic variant (T13240G, G12299A, 14058delG) in *TP53*. The recommendation from this report was that women with early-onset BC who had a family history consistent with LFS have a high probability of carrying a germline *TP53* pathogenic variant and that subgroups of families with early-onset BC might warrant *TP53* screening irrespective of meeting the Chompret and LFS criteria [14].

Bougeard et al. [37] detected *TP53* mutations (c.286_288del, c.991C > T, c.375G > A) in 3/45 (6.7%) women who were diagnosed with breast cancer under 33 years of age with negative family history.

In a Dutch family study, 180 patients were referred for *TP53* pathogenic variant screening and were evaluated. Probands were diagnosed with breast cancer < 30 years of age. A *TP53* germline variant was identified in 24 probands who had positive family histories of cancer: 18 variants were found in LFS and LFL families, 2 variants were detected outside the ‘Chompret group’—child with rhabdomyosarcoma and a young woman with breast cancer. This study also revealed that among the families of *TP53* pathogenic variant carriers in addition to typical LFS tumors, colorectal cancer and pancreatic cancer occurred more frequently than in the general Dutch population [38].

Similar to many other studies, the women carrying germline *TP53* pathogenic variants were diagnosed with HER2 positive tumours, three of which were scored at 3 + and one of them was judged as HER2 negative tumor (HER2 1 +).

One Asian study showed that 5/83 (6%) of BC patients (< 35 years old) carried germline pathogenic variants in *TP53*. The patients originatted from the three ethnic populations in Malaysia and included Malay, Chinese and Indian women. Variants were only detected among LFS/LFL families and six of the seven breast tumors (86%) in these women were associated with amplification of HER2. This suggests that HER2 positive receptor status may be a useful marker to identifying germline pathogen variants in *TP53* [8].

Eccles et al. [39] also classified patients for *TP53* screening taking into consideration HER2 status (score 3 +) from a large UK cohort study. Using this approach, 3% (9/304) of women were diagnosed with HER2-amplified BC ≤ 40 years of age. From 71 BC patients diagnosed under 31 years of age, five carried a pathogenic *TP53* variant and none had a history of LFS. While in a cohort of women with HER2 + BC diagnosed ≤ 50 years without any known family history, fulfilling the Chompret criteria, only 1/195 women carried a *TP53* pathogenic variant. The authors concluded that patients under 41 years of age with a HER2( +) BC with a negative family history of breast cancer can be reassured that they have a low probability of being a high-risk gene carrier. If, however, there is a strong family history, not only *BRCA1* and *BRCA2* but also *TP53* testing should be considered.

Among our mutation carriers, 3 out of 4 patients (75%) had HER2 (3 +) receptor expression, while among the cohort of 100 BC patients, 21 had a positive HER2 (3 +) status. These findings support the results from other studies, which show that in women diagnosed with HER2 positive breast cancer under the age of 30 years with a family history of LFS, indicating that HER2 amplification may be a useful marker in identifying *TP53* mutation carriers. Thus we estimate that in Polish population, *TP53* mutations can be found around 14% of HER2 positive BC diagnosed ≤ 31 years of age with a positive family history of LFS.

In conclusion, the prevalence of germline pathogenic *TP53* variants in early-onset breast cancer in Polish population appears not to be different from those in other populations. Given the 4% prevalence of the *TP53* mutations among Polish patients, we suggest that all patients who develop *BRCA*-negative breast cancer ≤ 30 years and have a positive family history of LFS-related cancers should be screened for *TP53* variants. In addition, patients with HER2 receptor positive tumours should be offered genetic counseling and genetic testing for mutations in *TP53* gene. Knowledge about the prevalence of germline pathogenic variants in *TP53* coupled with screening for these in appropriately selected women could be beneficial for patients and their families by initiating specific surveillance and treatment options for early cancer detection and/or prevention.

## Electronic supplementary material

Below is the link to the electronic supplementary material.

Supplementary file1 (PDF 176 kb)
