# Supplementary material for: Prevalence of germline TP53 variants among early-onset breast cancer patients from Polish population
Source: Breast Cancer. 2020 Sep 4;28(1):226–35. doi: 10.1007/s12282-020-01151-7 (PMC7796867; doi:10.1007/s12282-020-01151-7)
Supplement: Supplementary file 1 — Supplementary file1 (PDF 176 kb) [file 12282_2020_1151_MOESM1_ESM.pdf]

TP53	Primer pairs	Ta*	Amplicon size (bp)
exon 2-3	F: GTTGGAAGTGTCTCATGCTGGA / R: TAGATGGGTGAAAAGAGCAGTCAG	60.55 / 60.32	390
exon 3-4	F: GAGACCTGTGGGAAGCGAAAAT / R: CAACTTTGGGACAGGAGTCAGA	60.88 / 59.90	637
exon 5-6	F: CTCTCTAGCTCGCTAGTGGGTT / R: ATGGGGTTATAGGGAGGTCAAAT	61.01 / 58.68	624
exon 7	F: ACAGGTCTCCCCAAGG / R: AAACTGAGTGGGAGCAGTAAGGAGA	53.92 / 63.23	325
exon 8-9	F: CTCTCATCACATCCCCGGC / R: CAAATGCCCCAATTGCAGGTAA	59.93 / 60.03	634
exon 10	F: GTCAGCTGTATAGGTACTTGAAGT / R: CAGCTGCCTTTGACCATGAAG	57.79 / 59.80	356
exon 11	F: GTGGCCACCATCTTGATTTGAAT / R: GCAAGCAAGGGTTCAAAGACC	59.80 / 60.27	307

*Ta = Annealing temperature (^{o}C)
